# SCN4A p.R675Q Mutation Leading to Normokalemic Periodic Paralysis: A Family Report and Literature Review

**DOI:** 10.3389/fneur.2019.01138

**Published:** 2019-10-25

**Authors:** Jiejing Shi, Qianqian Qu, Haiyan Liu, Wenhao Cui, Yan Zhang, Haidong Lv, Zuneng Lu

**Affiliations:** ^1^Department of Neurology, Jiaozuo People's Hospital of Henan Province, Jiaozuo, China; ^2^Department of Neurology, Renmin Hospital of Wuhan University, Wuhan, China

**Keywords:** normokalemic periodic paralysis, SCN4A mutation, muscle imaging, muscle biopsy, pedigree

## Abstract

**Objective:** To investigate the clinical features, skeletal muscle imaging, and muscle pathological characteristics of normokalemic periodic paralysis (NormoKPP) caused by mutation of SCN4A gene p.R675Q.

**Methods:** The clinical data, skeletal muscle imaging, pathological data, and gene test results of a family with NormoKPP were collected in detail in October 2018. The previous literature was reviewed and used for comparative analysis.

**Results:** The proband was a 28-year-old male with paroxysmal weakness of both lower limbs for 14 years. Limb weakness was mainly manifested in the proximal extremities of both lower limbs, which occurred two to three times a year. The muscle weakness of each attack lasted for 1–2 weeks and gradually recovered. The blood potassium levels were normal. The abnormal signals of the posterior thigh muscle group and the medial calf muscle group could be seen on the magnetic resonance imaging (MRI) of the skeletal muscle, and the target-fiber could be seen in some muscle fibers in muscle pathology. The father of the proband and his brother had the same symptoms. In the same family, 10 people received genetic testing. The results showed that five had a mutation of SCN4A gene p.R675Q. The mutation gene came from the father of the proband.

**Conclusion:** NormoKPP is a clinically rare form of sodium ion channel disease. The clinical manifestations, skeletal muscle imaging, and pathological changes are different from the common hypokalemic periodic paralysis. SCN4A gene detection is an important means for the diagnosis of NormoKPP.

## Introduction

Periodic paralysis (PP) is an ion channel disease characterized by recurrent muscle weakness caused by mutations in the skeletal muscle ion channel gene. According to the level of potassium in the blood, it can be divided into hypokalemic, normokalemic, and hyperkalemic periodic paralysis (1). Among them, normokalemic periodic paralysis (NormoKPP) is the rarest subtype of PP. NomoKPP is an autosomal dominant hereditary disease. At present, there are more than 20 kinds of sodium channel alpha subunit SCN4A gene mutations reported, including R675G, R675Q, R675H, R675W, R1129Q, T704M, and M1592V. However, these mutations have also been reported to cause hypokalemic periodic paralysis and hyperkalemic periodic paralysis. The NormoKPP caused by the p.R675Q mutation of the skeletal muscle SCN4A gene has not been reported. In this study, the clinical features, muscle magnetic resonance imaging (MRI), and muscle pathology of a family case of NormoKPP was confirmed by genetic testing. We also conducted a thorough literature review.

## Materials and Methods

### Clinical Features

The proband was a 28-year-old male truck driver. The main complaint was “paroxysmal weakness of both lower limbs for 14 years, recurrence in February” in October 2018. About 14 years ago, the patient sweated after taking two tablets of Analgin orally because of a cold. In the morning, he developed limb weakness, with both lower limbs as the most important. He could not get out of bed or turn over. After about 1 week, the symptoms gradually improved and returned to normal. The effect of “low potassium” treatment in local hospitals is poor. Afterwards, the onset of muscle weakness lasted for 1–2 weeks, two to three times a year, and occurred mostly in the morning during summer. In most cases, it is still feasible to walk, but it is laborious to go up the stairs and some even cannot walk in serious cases. The blood potassium test was normal many times during the attack, and the symptoms of oral potassium chloride solution did not improve significantly. After a cold 2 months ago, limb weakness occurred again, mainly in the lower limbs; both upper limbs could be raised above the head, squatting and standing was difficult, and his ability to walk was weakened. The blood potassium was normal when measured at the local hospital. After 1 week, the proximal muscle strength of both lower extremities basically returned to normal, but the distal muscle strength of the left lower extremity did not completely return to normal. The symptoms of limb weakness still fluctuated in the past month. After walking for 100 m, the calves became sour and weak. So far, muscle strength has not completely recovered. He came to our hospital for treatment. He has a past of physical fitness. His father began to suffer from paroxysmal limb weakness in his teens, mostly in the morning with squatting and standing difficulties and walking weakness. It lasted for about a week, three to five times a year, and there had been no attack in nearly 10 years. His elder brother had intermittent weakness of both lower limbs since he was 16 years old, mainly in the calves, and he had difficulties standing on his tiptoes. He had an average attack of about two times a year. He had been tested for blood potassium in local hospitals many times. A total of four siblings, two sisters, and their mothers, were in good health and had no similar medical history. Physical examination: cranial nerve (–); normal limb muscle tension; upper limb muscle strength grade 5; lower limb proximal muscle strength grade 4; distal muscle strength grade 4+; tendon reflex of upper extremities (+); tendon reflex of lower extremities had disappeared; bilateral pathological signs (–); bilateral depth and shallow sensation were normal; extremities had no muscle atrophy and hypertrophy; there was no muscle tenderness.

## Methods

### Neuroelectrophysiology

Motor conduction velocity (MCV), sensory conduction velocity (SCV), and Concentric circular needle electromyography (EMG) were measured using the Japanese photoelectric MEB-9200K myoelectric evoked potential meter. Bilateral tibialis anterior, quadriceps femoris, gastrocnemius, and biceps brachii were selected for electromyography. The conduction velocity of the bilateral common peroneal nerve, tibial nerve, superficial peroneal nerve, and sural nerve was measured. There was parallel detection of F wave and H-reflex in both lower limbs.

### Muscle MRI

Axial scanning of the lower limbs of the patient was performed using a GE 1.0T nuclear magnetic resonance machine. The scanning sequences included T1WI, T2WI, and STIR.

### Muscle Pathology

The proband was given an open biopsy under local anesthesia, and the left gastrocnemius muscle was selected as the biopsy muscle. Muscle specimens were fixed by liquid nitrogen, frozen in sections, subjected to tissue HE, MGT, ORO, PAS, ATPase, and NADH staining, and observed under a light microscope.

### Genetic Testing

With the informed consent of the proband and his family members, genetic testing was performed on the proband and 10 families. Genomic DNA was extracted from 4 ml venous blood for screening of single gene genetic diseases of whole genome exons, which were detected by Jin Jun Inspection Center.

## Results

### Neuroelectrophysiology

SCV: The sensory nerves of both lower limbs were normal. MCV: The motor nerves of both lower limbs were normal. F wave: The F-waves of nerves detected in both lower limbs were normal. H-reflex: The H-reflex amplitude of the examined nerves in the left lower limb was lower than that in the opposite side, and the right side was normal. EMG: The muscles of the upper and lower limbs were normal. However, the patient failed to perform a long exercise test (2).

### Laboratory Examination

CK 380 μ/L, CKMB 26 μ/L, LDH 185 μ/L, LAC 2.31 mmol/L, ALT 27 μ/L, AST 23 μ/L, K^+^ 4.31 mmol/L, Ca2^+^ 2.34 mmol/L, Na^+^ 142 mmol/L, and CL^−^ 105 mmol/L.

### MRI Scan

**Figure 1:**
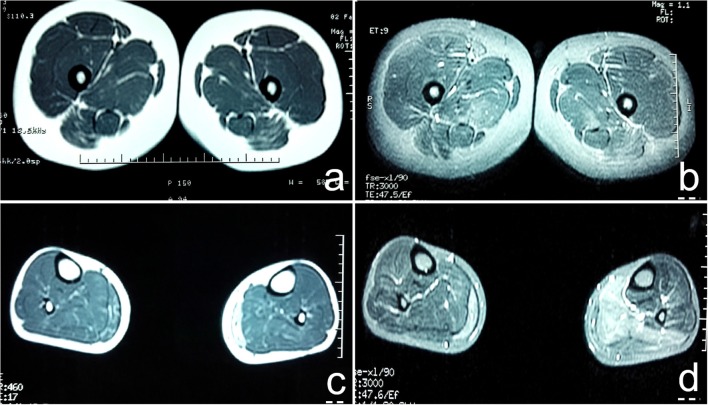
**(a)** T1WI sequence showed diffuse increase in signal intensity of bilateral gluteus maximus, gluteus medius, adductor maximus, and sartorius muscles; **(b)** T2W1 with fat suppression showed multiple high signal intensity in strips and lines in bilateral gluteus maximus, gluteus medius, adductor maximus, and sartorius muscles; **(c)** T1WI showed diffuse increase in signal intensity of medial head of bilateral gastrocnemius, present reduction of muscle volume. The medial head of the lateral gastrocnemius was obvious; **(d)** T2W1 with fat suppression showed that the signal intensity of the medial head of the right gastrocnemius and the posterior leg muscle group increased unevenly, especially the medial head of the left gastrocnemius.

The muscle groups of bilateral gluteus maximus, bilateral gluteus medius, bilateral adductor maximus, and bilateral sartorius, the medial head of left gastrocnemius and the posterior leg showed diffuse increase signals and present reduction of muscle volume (Figure 1).

### Muscle Pathology

**Figure 2:**
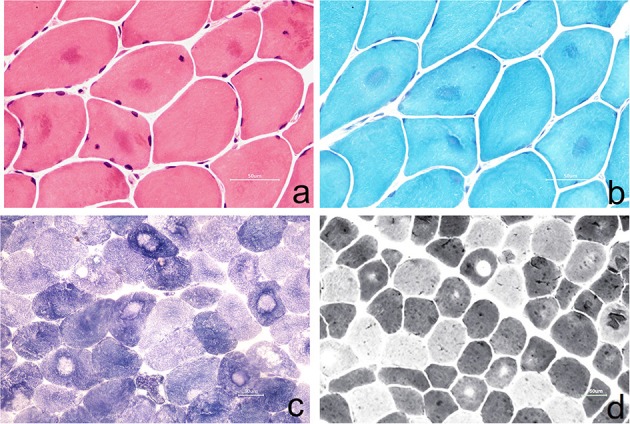
**(a)** Muscle fibers vary slightly in size, with round-like changes. Some of them show eosinophilic deep-stained areas in the center of muscle fibers (HE staining, × 400); **(b)** some muscle fibers were dyed dark blue in the center of the by MGT staining, and no typical RRF were observed (MGT staining, × 400); **(c)** the arrangement of myofibrils in some muscle fibers was disordered, and the absence of activity in the center of muscle fibers resulted in target-fiber (NADH staining × 200); **(d)** the distribution of type I and type II muscle fibers was basically normal, and the phenomenon of target-fiber appeared mostly in type II muscle fibers (ATPase staining × 200).

The left gastrocnemius muscle biopsy was performed with the consent of the patient (Figure 2).

### Genetic Testing

**Figure 3:**
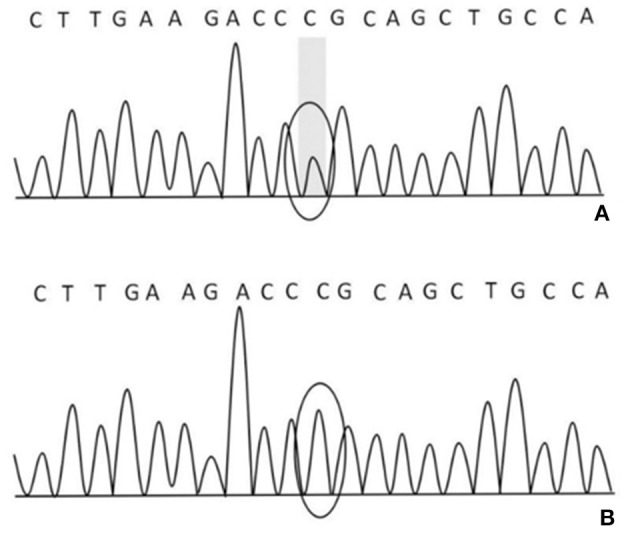
**(A)** There was a heterozygous mutation c.2024 4>A in the proband, his father, brother, son, and daughter on chr17-62034874; **(B)** there was no mutation in his mother, son, the eldest sister's son, the second sister's daughter, and his brother's daughter on chr17-62034874.

**Figure 4:**
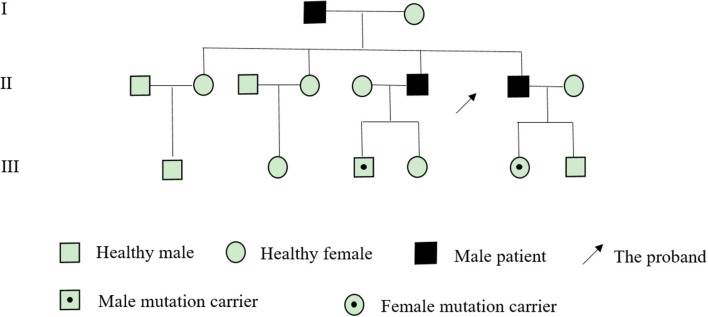
Pedigree of a family presenting with NormoKPP.

After obtaining the consent of the probands and their families, 10 pedestrians were tested for the probands and their families. The results showed that there was a heterozygous mutation in the patient-related gene SCN4A: c.2024 4 > A (guanine > adenine), which led to the change of amino acid p.R675Q. Family validation results showed that the heterozygous mutation results came from the father, and there were the same heterozygous mutations at this locus in his brother, his son, and his daughter (Figure 3). There were no mutations in the genes of her mother, her son, her eldest sister's son, her second sister's daughter, and her brother's daughter. Other relatives did not receive genetic testing (Figure 4).

## Discussion

NormoKPP is a rare type of PP. Patients often develop symptoms around the age of 10, which is characterized by paroxysmal muscle weakness, without changes in serum potassium concentration. Each attack of limb weakness lasts for a relatively long time, usually several days to weeks, before returning to normal. Most of the symptoms are alleviated in adulthood while a few can be left with persistent muscle weakness and muscle atrophy (1). In this family, all three patients had onset in adolescence, limb weakness mainly involved both lower limbs, and both proximal and distal muscles were involved. After the attack, the clinical symptoms recovered slowly, and the muscle weakness symptoms lasted for a long time. In most cases, the symptoms gradually recovered after 1–2 weeks. Both the proband and his brother had a history of distal limb weakness lasting for more than 4 weeks, which has not been reported in previous literature (3–9).

There are different degrees of high signal on fat-suppressed T2WI. The most vulnerable muscles are the soleus, anterior, lateral head of gastrocnemius, and medial head of gastrocnemius (10). However, there are few studies on skeletal muscle imaging in patients with NormoKPP, and the literature is limited. In this case, the patient's leg MRI showed a diffuse increase in the bilateral gluteus maximus, gluteus medius, adductor magnus, and sartorius in the STIR sequence, which is suggestive of muscle edema. The STIR and T2WI sequence showed diffuse and uneven increase of signal intensity in the medial head of the right gastrocnemius and the posterior calve muscle group, significant in the medial head of left gastrocnemius, suggesting muscle edema and partial fatization. In T1WI, there were multiple cases of linear high signal intensity in the above muscles, diffuse increase of signal intensity in the medial head of left gastrocnemius, and present reduction of muscle volume; the medial head of the bilateral gastrocnemius was obvious, suggesting that the muscles of the lower limbs of the patient were replaced by fat and had mild muscle atrophy. This has not been reported in the literature.

Muscle biopsy in NormoKPP patients is also less reported (3–9). It has been reported that pathological findings include the enlargement of the sarcoplasmic reticulum, an increase of mitochondria, accumulation of myotubes, and focal myofibrillar necrosis in persistent limb weakness (11). In this case, a muscle biopsy showed that the muscle fibers were slightly different in size with round-like changes. In the center of some muscle fibers, eosinophilic deep-stained areas were seen, and no obvious infiltration of denatured and necrotic muscle fibers and inflammatory cells was observed. In nicotinamide adenine dinucleotidehydrogen (NADH) staining, the arrangement of myofibrils in some muscle fibers was disordered, and the absence of activity in the center of the muscle fibers resulted in target-fiber, which was seldom reported in previous literature. The modified Gomoritrichrome (MGT) staining showed that some muscle fibers were dyed dark blue in the center, consistent with the NADH staining, and no typical tubular aggregation and ragged red fibers (RRF) were observed. ATPase staining showed that the distribution of type I and II muscle fibers was basically normal, and the phenomenon of target-fiber appeared mostly in type II muscle fibers. However, the pathological changes mentioned above are rarely reported in the previous literature.

The SCN4A gene is located on the 17q23-25 chromosome (11), and its mutation can lead to various types of periodic paralysis. R675Q mutation is located in the DIIS4 region of the sodium channel. This mutation may increase the continuous current of the sodium channel, change the voltage-dependent activation process, lead to abnormal depolarization of resting potential, slow down the inactivation process, thus affecting the normal function of sodium channel, and lead to disease (11). The genetic testing results of this family suggest that the mutation of R675Q in the SCN4A gene comes from the father, and the brother also carries the gene. Compared with the previously reported cases of NormoKPP, the familial NormoKPP caused by the mutation of p.R675Q in the SCN4A gene has not been reported (7, 8, 12). The clinical features of such patients were long duration of muscle weakness symptoms and slow recovery of muscle strength. However, with the increase of age, the number of seizures gradually decreased or terminated. Skeletal muscle MRI is characterized by abnormal signals of the posterior thigh muscles and the medial calf muscles. Muscle pathology showed that some of the muscle fibers have a target-fiber phenomenon.

It has been reported that most of the gene mutations of NormoKPP are common to HyperKPP (13, 14). Therefore, in clinical practice, we should make a comprehensive analysis based on clinical characteristics and biochemical detection, combined with skeletal muscle imaging and pathological changes and genetic testing results to determine the final clinical diagnosis. But, why the same gene mutation of SCN4A causes PP of different types of serum potassium levels still needs further study.

## Ethics Statement

The studies involving human participants were reviewed and approved by the ethics committee of the Jiaozuo People's Hospital of Henan Province, Henan, China. The patients/participants provided their written informed consent to participate in this study.

## Author Contributions

JS was responsible for writing papers. QQ and HLi are responsible for data collection. WC was responsible for making important revisions to papers. YZ was responsible for data analysis. HLv and ZL are responsible for providing overall ideas.

### Conflict of Interest

The authors declare that the research was conducted in the absence of any commercial or financial relationships that could be construed as a potential conflict of interest.
